# Independent and joint associations of intrinsic capacity and frailty index with incident disability and all-cause mortality among older Chinese adults

**DOI:** 10.3389/fpubh.2026.1859572

**Published:** 2026-09-16

**Authors:** Bing Feng, Ji Wang, Yaowen Li, Liangliang Ma, Yue Yin, Juan Xu, Yuhan Dong

**Affiliations:** 1 Department of Traditional Chinese Medicine Rehabilitation, The 966th Hospital of The PLA Joint Logistic Support Force of China, Dandong, Liaoning, China; 2 Outpatient Department of the 15th Veteran Cadre Sanatorium of Beijing Garrison Command, Beijing, China; 3 Outpatient Department, Liaoning Provincial Military District Dandong First Retired Cadres Sanatorium, Dandong, Liaoning, China; 4 Department of General Surgery, Affiliated Xiaoshan Hospital, Hangzhou Normal University, Hangzhou, China

**Keywords:** disability, frailty, intrinsic capacity, mortality, older adults

## Abstract

**Background:**

Intrinsic capacity (IC) and the frailty index (FI) constitute two conceptually distinct frameworks for evaluating health status in later life; however, their comparative and combined prognostic utility for incident disability and all-cause mortality has not been fully elucidated.

**Methods:**

This prospective cohort study included 7,168 adults aged ≥60 years from the 2014 wave of the Chinese Longitudinal Healthy Longevity Survey. IC was evaluated across five domains and aggregated into a composite score ranging from 0 to 10. FI was derived using a deficit accumulation approach. Incident disability was defined as new-onset limitation in at least one of six basic activities of daily living in 2018. All-cause mortality was ascertained during follow-up. Multivariable logistic regression and Cox proportional hazards models were applied, together with joint association and incremental predictive analyses.

**Results:**

In fully adjusted models, higher IC was independently associated with lower odds of incident disability [odds ratio (OR) = 0.79, 95% confidence interval (CI): 0.74–0.84], whereas higher FI was associated with greater odds of disability (OR per 0.1 increase = 1.93, 95% CI: 1.67–2.23). Similarly, higher IC was associated with reduced risk of all-cause mortality [hazard ratio (HR) = 0.84, 95% CI: 0.82–0.85], whereas higher FI was associated with increased mortality risk (HR per 0.1 increase = 1.25, 95% CI: 1.19–1.31). After mutual adjustment, both IC and FI remained significantly associated with incident disability, whereas only IC remained significantly associated with all-cause mortality. Participants with concomitantly lower IC and higher FI exhibited the greatest risks of both disability (OR = 4.31, 95% CI: 2.65–7.04) and mortality (HR = 2.66, 95% CI: 2.32–3.04).

**Conclusion:**

Both IC and FI were significantly associated with incident disability and all-cause mortality among older Chinese adults. The combination of IC and FI identified distinct profiles of functional and mortality risk, while IC showed a stronger association with mortality than FI.

## Introduction

Population aging is accelerating globally, rendering the preservation of health and functional independence in later life an increasingly important public health priority (1). Among older adults, disability and mortality represent two of the most consequential adverse outcomes because both are strongly associated with loss of autonomy, escalating care requirements, and increased healthcare burden (2–4). Disability in activities of daily living (ADL), in particular, frequently signifies a transition from independent living to sustained dependency, whereas mortality remains the most definitive endpoint in geriatric research (5, 6). Accordingly, early identification of older individuals at elevated risk of disability and death is essential for effective risk stratification and preventive intervention.

Intrinsic capacity (IC) and the frailty index (FI) are two widely used yet conceptually distinct frameworks for evaluating health status in older adults. Introduced within the World Health Organization framework of healthy aging, IC reflects the composite of an individual’s physical and mental capacities (7). In contrast, FI is based on the cumulative deficit model and quantifies the burden of multidimensional health deficits (8). Although both measures have been linked to adverse outcomes in older populations (9, 10), they are not interchangeable constructs. IC is intended to characterize preserved function and physiologic reserve, whereas FI reflects accumulated deficits and vulnerability (11–13). Clarifying how these two indicators relate to major geriatric outcomes may help determine whether they capture overlapping or complementary prognostic information.

However, prior studies have predominantly examined IC and frailty in isolation, and direct comparative evaluations remain limited (14–16). In particular, few investigations have assessed their independent associations, joint effects, and incremental predictive value for both incident disability and all-cause mortality within the same cohort. This knowledge gap is especially relevant in older Chinese populations, in whom the burden of disability is substantial and practical tools for risk stratification are needed (17). Furthermore, because IC is inherently multidimensional, the extent to which its individual domains differentially contribute to the risks of disability and mortality remains incompletely understood (14, 18).

Using data from the Chinese Longitudinal Healthy Longevity Survey, this study examined the associations of IC and FI with incident disability and all-cause mortality among adults aged 60 years or older. Mutually adjusted associations, joint associations, and the associations of the five IC domains with these outcomes were explored. It was hypothesized that lower IC and higher FI would each be associated with increased risks of incident disability and all-cause mortality, but that their relative contributions to these outcomes would differ.

## Materials and methods

### Study population and participant selection

This study used data from the 2014 wave of the Chinese Longitudinal Healthy Longevity Survey (CLHLS), a nationwide prospective cohort study of older adults in China. Data were collected through face-to-face interviews using standardized questionnaires. Detailed descriptions of the CLHLS have been published previously.

Among 7,192 participants enrolled in 2014, 7,168 individuals aged ≥60 years were included in the present analysis. For analyses of incident disability, participants with missing baseline ADL information, those with baseline disability, and those lacking disability information in 2018 were excluded. For analyses of all-cause mortality, participants without mortality status or survival time data were excluded. Because eligibility additionally depended on the availability of exposure and covariate data, the final analytical sample varied across statistical models. The participant selection process is illustrated in Supplementary Figure S1.

### Assessment of intrinsic capacity

IC was assessed across five domains: locomotion, sensory function, vitality, psychosocial function, and cognition (12). Detailed definitions and coding criteria are provided in Supplementary Table 1.

Briefly, locomotion was assessed by walking ability; sensory function by hearing and vision; vitality by the ability to lift a 5-kg weight; psychosocial function by five items reflecting emotional and psychological status; and cognition by the Chinese version of the Mini-Mental State Examination. Each domain was scored from 0 to 2, with higher scores indicating better function. The total IC score ranged from 0 to 10 and was analyzed as both a continuous and categorical variable. For categorical analyses, IC was categorized into quartiles. For joint analyses, IC was dichotomized as the lowest quartile versus the upper three quartiles.

### Construction of the frailty index

Frailty was assessed using a deficit accumulation approach (8). A FI was constructed from 37 health deficits spanning multiple domains, including chronic diseases, functional status, self-rated health, psychological status, sensory problems, and physical conditions (19). The complete list of items and coding criteria is provided in Supplementary Table 1. Each deficit was coded on a scale from 0 to 1, with higher values indicating poorer health status. The FI was calculated as the sum of deficit scores divided by the number of non-missing items and was computed only for participants with at least 30 available items. The final FI ranged from 0 to 1, with higher values indicating greater frailty. In the primary analyses, FI was modeled as both a continuous and categorical variable. For continuous analyses, FI was analyzed per 0.1-point increment. For categorical analyses, participants were classified as robust (FI ≤ 0.10), pre-frail (FI > 0.10 to ≤0.21), or frail (FI > 0.21) (20). For joint analyses, FI was further dichotomized as non-frail (FI ≤ 0.21) versus frail (FI > 0.21).

### Covariates

Covariates were selected *a priori* based on previous literature regarding factors associated with intrinsic capacity, frailty, functional disability, and mortality among older adults, as well as data availability. The selected covariates included age, sex, smoking status, alcohol drinking status, residence, education level, marital status, economic status, self-rated health, self-rated quality of life, hypertension, diabetes, stroke, and heart disease. These variables were considered potential confounding factors or factors associated with the study outcomes.

Because several health-related variables were also incorporated into the FI, distinct adjustment sets were applied across models. IC-only models were adjusted for age, sex, smoking status, alcohol drinking status, residence, education level, marital status, economic status, self-rated health, self-rated quality of life, hypertension, diabetes, stroke, and heart disease. FI-only and joint IC-FI models were adjusted for age, sex, smoking status, alcohol drinking status, residence, education level, marital status, economic status, and self-rated quality of life.

### Outcome assessment

Incident disability was defined using six basic ADL: bathing, dressing, toileting, indoor transferring, continence, and eating. For each item, participants were asked whether they had experienced limitation for at least the preceding 6 months because of a health problem. Any report of requiring assistance was classified as a limitation. Participants reporting at least one limitation across the six domains were considered to have ADL disability. For the incident disability analysis, only participants who were free of ADL disability at baseline, survived to the 2018 follow-up, and had available ADL information in 2018 were included. Participants who died before the 2018 follow-up were not assigned a disability status because follow-up ADL could not be assessed. Incident disability was defined as new-onset disability in 2018.

All-cause mortality was ascertained during follow-up through face-to-face interviews conducted as part of the CLHLS. Vital status and date of death were obtained from family members or other informed respondents when a participant had died. Survival time was calculated from baseline in 2014 until death or the last follow-up.

### Statistical analysis

Baseline characteristics were described according to quartiles of IC and categories of FI. Continuous variables are presented as mean ± standard deviation, and categorical variables as number (%). Group differences were compared using analysis of variance or the chi-square test, as appropriate. Interaction analyses between IC and FI were conducted on both multiplicative and additive scales. Multivariable logistic regression was used to examine the associations of IC and FI with incident disability, whereas Cox proportional hazards regression was used to evaluate associations with all-cause mortality. IC and FI were first analyzed separately, then as categorical exposures, and finally simultaneously in mutually adjusted models. Three models were fitted: Model 1, unadjusted; Model 2, adjusted for age and sex; and Model 3, fully adjusted. To assess joint associations, IC and FI were combined into four groups: higher IC + lower FI, higher IC + higher FI, lower IC + lower FI, and lower IC + higher FI, with the higher IC + lower FI group serving as the reference. Logistic and Cox regression models were used to estimate associations with incident disability and all-cause mortality, respectively. Kaplan–Meier curves were compared using the log-rank test, and *post hoc* pairwise comparisons between groups were performed with adjustment for multiple comparisons. Restricted cubic spline analyses were conducted to explore potential nonlinear associations between continuous IC/FI scores and study outcomes, with detailed modeling procedures provided in the Supplementary material. Domain-specific analyses were additionally conducted for the five IC domains. Several sensitivity analyses were performed to assess the robustness of the findings, with detailed definitions and analytical procedures provided in the Supplementary material. Details of the restricted cubic spline analyses are also provided in the Supplementary material. All analyses were performed using Stata version 18.0, and a two-sided *p* value <0.05 was considered statistically significant.

## Results

### Baseline characteristics

Baseline characteristics according to quartiles of IC score are presented in Table 1, and baseline characteristics according to FI categories are presented in Table 2. Significant differences across IC quartiles and FI categories were observed for age, sociodemographic characteristics, self-rated health, self-rated quality of life, and chronic disease history (all *p* < 0.05). Supplementary Tables 2 and 3 further compare participants included in versus excluded from the final joint IC-FI Model 3 for incident disability and all-cause mortality, respectively. Supplementary Table 4 additionally presents baseline characteristics according to dichotomized IC and FI categories.

**Table 1:** Baseline characteristics of the participants according to quartiles of intrinsic capacity score.

Variable	Overall (*N* = 6,411)	Q1 (*N* = 1,736)	Q2 (*N* = 1,485)	Q3 (*N* = 2,053)	Q4 (*N* = 1,137)	*p* value
Age, years	85.23 ± 10.55	93.88 ± 8.92	86.89 ± 8.91	80.82 ± 8.51	77.84 ± 8.02	<0.001
Sex						<0.001
Male	2,968 (46.3)	511 (29.4)	595 (40.1)	1,113 (54.2)	749 (65.9)	
Female	3,443 (53.7)	1,225 (70.6)	890 (59.9)	940 (45.8)	388 (34.1)	
Residence						<0.001
Rural	3,500 (54.6)	1,015 (58.5)	796 (53.6)	1,140 (55.5)	549 (48.3)	
Town	2029 (31.6)	504 (29.0)	484 (32.6)	676 (32.9)	365 (32.1)	
City	882 (13.8)	217 (12.5)	205 (13.8)	237 (11.5)	223 (19.6)	
Education level, years						<0.001
<=5	5,131 (80.6)	1,588 (92.4)	1,281 (86.8)	1,550 (75.9)	712 (63.0)	
>5	1,234 (19.4)	130 (7.6)	194 (13.2)	492 (24.1)	418 (37.0)	
Marital status						<0.001
Unmarried/divorced/widowed	3,797 (59.8)	1,412 (82.1)	1,019 (69.3)	1,000 (49.1)	366 (32.6)	
Married	2,552 (40.2)	308 (17.9)	451 (30.7)	1,036 (50.9)	757 (67.4)	
Economic status						<0.001
Poor	735 (11.6)	304 (18.1)	191 (13.0)	188 (9.2)	52 (4.6)	
Average	4,555 (72.0)	1,192 (71.2)	1,061 (72.1)	1,488 (72.7)	814 (71.8)	
Rich	1,037 (16.4)	179 (10.7)	220 (14.9)	371 (18.1)	267 (23.6)	
Smoking status						<0.001
Never	4,457 (69.8)	1,412 (81.7)	1,055 (71.5)	1,345 (65.6)	645 (57.0)	
Former	856 (13.4)	202 (11.7)	192 (13.0)	275 (13.4)	187 (16.5)	
Current	1,072 (16.8)	115 (6.7)	228 (15.5)	429 (20.9)	300 (26.5)	
Alcohol drinking status						<0.001
Never	4,767 (74.9)	1,453 (84.2)	1,120 (76.0)	1,478 (72.7)	716 (63.4)	
Former	632 (9.9)	159 (9.2)	159 (10.8)	189 (9.3)	125 (11.1)	
Current	963 (15.1)	114 (6.6)	194 (13.2)	366 (18.0)	289 (25.6)	
Self-rated health						<0.001
Poor/very poor	933 (15.4)	397 (28.1)	298 (20.4)	185 (9.0)	53 (4.7)	
Fair	2,428 (40.1)	608 (43.0)	643 (44.0)	849 (41.4)	328 (28.9)	
Good/very good	2,701 (44.6)	408 (28.9)	520 (35.6)	1,018 (49.6)	755 (66.5)	
Self-rated quality of life						<0.001
Poor/very poor	227 (3.7)	121 (8.5)	58 (4.0)	41 (2.0)	7 (0.6)	
Fair	1796 (29.6)	484 (34.2)	451 (30.8)	602 (29.4)	259 (22.8)	
Good/very good	4,045 (66.7)	812 (57.3)	954 (65.2)	1,408 (68.6)	871 (76.6)	
Hypertension						<0.001
No	3,971 (63.4)	1,106 (65.6)	859 (59.3)	1,294 (64.5)	712 (63.3)	
Unknown	239 (3.8)	126 (7.5)	44 (3.0)	49 (2.4)	20 (1.8)	
Yes	2053 (32.8)	454 (26.9)	545 (37.6)	662 (33.0)	392 (34.9)	
Diabetes						<0.001
No	5,594 (90.1)	1,457 (86.8)	1,295 (90.6)	1819 (91.3)	1,023 (92.2)	
Unknown	262 (4.2)	146 (8.7)	50 (3.5)	46 (2.3)	20 (1.8)	
Yes	354 (5.7)	75 (4.5)	84 (5.9)	128 (6.4)	67 (6.0)	
Stroke						<0.001
No	5,157 (83.0)	1,345 (80.3)	1,158 (80.9)	1,680 (84.3)	974 (87.7)	
Unknown	245 (3.9)	129 (7.7)	53 (3.7)	42 (2.1)	21 (1.9)	
Yes	809 (13.0)	202 (12.1)	221 (15.4)	271 (13.6)	115 (10.4)	
Heart disease						<0.001
No	5,427 (87.4)	1,359 (80.9)	1,243 (86.6)	1797 (90.2)	1,028 (93.2)	
Unknown	231 (3.7)	124 (7.4)	53 (3.7)	37 (1.9)	17 (1.5)	
Yes	553 (8.9)	197 (11.7)	139 (9.7)	159 (8.0)	58 (5.3)	
Frailty index	0.19 ± 0.10	0.27 ± 0.11	0.20 ± 0.09	0.14 ± 0.07	0.12 ± 0.06	<0.001

Continuous variables are presented as mean ± SD, and categorical variables are presented as *n* (%). *p* values were calculated using one-way ANOVA for continuous variables and chi-square tests for categorical variables. Percentages were calculated based on non-missing data for each variable.

**Table 2:** Baseline characteristics of the participants according to frailty index categories.

Variable	Overall (*N* = 6,734)	Robust (*N* = 1,345)	Pre-frail (*N* = 3,118)	Frail (*N* = 2,271)	*p* value
Age, years	85.38 ± 10.63	80.54 ± 9.71	84.98 ± 10.16	88.80 ± 10.57	<0.001
Sex					<0.001
Male	3,087 (45.8)	808 (60.1)	1,437 (46.1)	842 (37.1)	
Female	3,647 (54.2)	537 (39.9)	1,681 (53.9)	1,429 (62.9)	
Residence					<0.001
Rural	3,688 (54.8)	771 (57.3)	1811 (58.1)	1,106 (48.7)	
Town	2,121 (31.5)	422 (31.4)	936 (30.0)	763 (33.6)	
City	925 (13.7)	152 (11.3)	371 (11.9)	402 (17.7)	
Education level, years					<0.001
<=5	5,365 (80.4)	951 (71.0)	2,504 (81.2)	1910 (84.7)	
>5	1,312 (19.6)	388 (29.0)	578 (18.8)	346 (15.3)	
Marital status					<0.001
Unmarried/divorced/widowed	4,004 (60.1)	585 (44.1)	1848 (59.8)	1,571 (70.0)	
Married	2,654 (39.9)	741 (55.9)	1,240 (40.2)	673 (30.0)	
Economic status					<0.001
Poor	747 (11.3)	65 (4.9)	304 (9.8)	378 (17.1)	
Average	4,812 (72.5)	971 (72.6)	2,296 (74.3)	1,545 (70.0)	
Rich	1,076 (16.2)	302 (22.6)	491 (15.9)	283 (12.8)	
Smoking status					<0.001
Never	4,708 (70.2)	845 (63.1)	2,172 (70.0)	1,691 (74.8)	
Former	883 (13.2)	137 (10.2)	402 (13.0)	344 (15.2)	
Current	1,112 (16.6)	358 (26.7)	528 (17.0)	226 (10.0)	
Alcohol drinking status					<0.001
Never	5,017 (75.1)	926 (69.4)	2,334 (75.5)	1757 (78.0)	
Former	645 (9.7)	74 (5.5)	282 (9.1)	289 (12.8)	
Current	1,015 (15.2)	334 (25.0)	474 (15.3)	207 (9.2)	
Self-rated health					<0.001
Poor/very poor	935 (14.9)	13 (1.0)	246 (8.1)	676 (35.4)	
Fair	2,519 (40.1)	309 (23.0)	1,348 (44.5)	862 (45.1)	
Good/very good	2,827 (45.0)	1,022 (76.0)	1,433 (47.3)	372 (19.5)	
Self-rated quality of life					<0.001
Poor/very poor	222 (3.5)	10 (0.7)	69 (2.3)	143 (7.5)	
Fair	1837 (29.2)	279 (20.8)	874 (28.9)	684 (35.8)	
Good/very good	4,227 (67.2)	1,055 (78.5)	2086 (68.9)	1,086 (56.8)	
Hypertension					<0.001
No	4,303 (64.0)	1,158 (86.2)	2034 (65.4)	1,111 (49.0)	
Unknown	272 (4.0)	8 (0.6)	55 (1.8)	209 (9.2)	
Yes	2,148 (32.0)	178 (13.2)	1,021 (32.8)	949 (41.8)	
Diabetes					<0.001
No	6,064 (90.1)	1,329 (98.8)	2,952 (94.8)	1783 (78.6)	
Unknown	301 (4.5)	2 (0.1)	41 (1.3)	258 (11.4)	
Yes	362 (5.4)	14 (1.0)	120 (3.9)	228 (10.0)	
Stroke					<0.001
No	5,592 (83.2)	1,315 (97.8)	2,788 (89.5)	1,489 (65.9)	
Unknown	277 (4.1)	3 (0.2)	34 (1.1)	240 (10.6)	
Yes	850 (12.7)	27 (2.0)	293 (9.4)	530 (23.5)	
Heart disease					<0.001
No	5,888 (87.5)	1,335 (99.3)	2,933 (94.1)	1,620 (71.6)	
Unknown	261 (3.9)	1 (0.1)	22 (0.7)	238 (10.5)	
Yes	577 (8.6)	9 (0.7)	162 (5.2)	406 (17.9)	
Intrinsic capacity score	6.41 ± 3.10	8.68 ± 1.57	7.06 ± 2.57	4.12 ± 3.03	<0.001

Continuous variables are presented as mean ± SD, and categorical variables are presented as *n* (%). *p* values were calculated using one-way ANOVA for continuous variables and chi-square tests for categorical variables. Percentages were calculated based on non-missing data for each variable.

### Associations of IC and FI with incident disability

Among the 2,799 participants eligible for the incident disability analysis, 474 participants developed incident disability during follow-up, corresponding to a cumulative incidence of 16.9%. Table 3 presents the associations of IC and FI with incident disability from 2014 to 2018. In the fully adjusted model, higher IC was associated with lower odds of incident disability (OR = 0.79, 95% CI: 0.74–0.84), whereas higher FI was associated with greater odds of incident disability (OR per 0.1 increase = 1.93, 95% CI: 1.67–2.23). Relative to the reference groups, participants in the lowest IC quartile had the highest odds of incident disability (OR = 4.62, 95% CI: 2.77–7.69), whereas robust participants had lower odds than frail participants (OR = 0.19, 95% CI: 0.12–0.29). When IC and FI were entered simultaneously into the model, both remained significantly associated with incident disability, although effect estimates were attenuated. Restricted cubic spline analyses revealed that both IC and FI were significantly associated with incident disability (overall *p* < 0.001 for both), with no evidence of nonlinearity (P for nonlinearity = 0.267 for IC and 0.153 for FI; Supplementary Figure S2). Domain-specific analyses are presented in Supplementary Table 5.

**Table 3:** Associations of intrinsic capacity and frailty index with incident disability from 2014 to 2018.

Variables	Model 1		Model 2		Model 3	
	OR (95% CI)	*p* value	OR (95% CI)	*p* value	OR (95% CI)	*p* value
Part A. Intrinsic capacity and incident disability (*N* = 2,387)						
IC score, per 1-point increase	0.74 (0.70, 0.77)	<0.001	0.83 (0.79, 0.88)	<0.001	0.79 (0.74, 0.84)	<0.001
IC quartiles (Q4 as reference)						
Q3	1.89 (1.36, 2.61)	<0.001	1.54 (1.11, 2.16)	0.011	1.50 (1.05, 2.14)	0.024
Q2	4.58 (3.27, 6.42)	<0.001	2.82 (1.97, 4.03)	<0.001	2.80 (1.89, 4.15)	<0.001
Q1	8.83 (5.93, 13.14)	<0.001	3.38 (2.17, 5.27)	<0.001	4.62 (2.77, 7.69)	<0.001
Part B. Frailty index and incident disability (*N* = 2,573)						
FI, per 0.1 increase	1.89 (1.68, 2.13)	<0.001	1.78 (1.57, 2.03)	<0.001	1.93 (1.67, 2.23)	<0.001
FI categories (frail as reference)						
Pre-frail	0.58 (0.46, 0.72)	<0.001	0.60 (0.47, 0.77)	<0.001	0.57 (0.44, 0.74)	<0.001
Robust	0.15 (0.10, 0.22)	<0.001	0.20 (0.14, 0.31)	<0.001	0.19 (0.12, 0.29)	<0.001
Part C. Mutually adjusted associations of intrinsic capacity and frailty index with incident disability (*N* = 2,394)						
IC score, per 1-point increase	0.78 (0.74, 0.82)	<0.001	0.90 (0.85, 0.95)	<0.001	0.84 (0.79, 0.90)	<0.001
FI, per 0.1 increase	1.42 (1.23, 1.64)	<0.001	1.62 (1.39, 1.88)	<0.001	1.69 (1.43, 2.00)	<0.001

Model 1 was unadjusted. Model 2 was adjusted for age and sex. Model 3 in Part A was additionally adjusted for smoking status, alcohol drinking status, residence, education level, marital status, economic status, self-rated health, self-rated quality of life, hypertension, diabetes, stroke, and heart disease. Model 3 in Parts B and C was additionally adjusted for smoking status, alcohol drinking status, residence, education level, marital status, economic status, and self-rated quality of life. FI categories were coded with frail as the reference group.

### Associations of intrinsic capacity and frailty index with all-cause mortality

Among the 5,648 participants eligible for the all-cause mortality analysis, 2,225 participants died from any cause during follow-up, corresponding to a cumulative mortality of 39.4%. Table 4 presents the associations of IC and FI with all-cause mortality. In the fully adjusted IC-only model, higher IC was associated with lower risk of all-cause mortality (HR = 0.84, 95% CI: 0.82–0.85). In the fully adjusted FI-only model, higher FI was associated with increased mortality risk (HR per 0.1 increase = 1.25, 95% CI: 1.19–1.31). However, when IC and FI were entered simultaneously, IC remained significantly associated with all-cause mortality (HR = 0.82, 95% CI: 0.81–0.84), whereas FI was no longer statistically significant (HR = 0.97, 95% CI: 0.91–1.03). Restricted cubic spline analyses showed that both IC and FI were significantly associated with mortality (overall *p* < 0.001 for both), and no evidence of nonlinearity was found (P for nonlinearity = 0.518 and 0.112, respectively; Supplementary Figure S3). Domain-specific results are presented in Supplementary Table 6.

**Table 4:** Associations of intrinsic capacity and frailty index with all-cause mortality.

Variables	Model 1		Model 2		Model 3	
	HR (95% CI)	*p* value	HR (95% CI)	*p* value	HR (95% CI)	*p* value
Part A. Intrinsic capacity and all-cause mortality (*N* = 4,474)						
IC score, per 1-point increase	0.77 (0.75, 0.78)	<0.001	0.82 (0.81, 0.84)	<0.001	0.84 (0.82, 0.85)	<0.001
IC quartiles (Q4 as reference)						
Q3	1.63 (1.33, 2.00)	<0.001	1.49 (1.22, 1.83)	<0.001	1.37 (1.11, 1.70)	0.004
Q2	3.89 (3.20, 4.74)	<0.001	2.94 (2.40, 3.59)	<0.001	2.57 (2.08, 3.19)	<0.001
Q1	9.70 (8.05, 11.69)	<0.001	5.38 (4.39, 6.60)	<0.001	4.19 (3.34, 5.26)	<0.001
Part B. Frailty index and all-cause mortality (*N* = 4,809)						
FI, per 0.1 increase	1.53 (1.47, 1.59)	<0.001	1.36 (1.30, 1.41)	<0.001	1.25 (1.19, 1.31)	<0.001
FI categories (frail as reference)						
Pre-frail	0.44 (0.40, 0.48)	<0.001	0.67 (0.58, 0.76)	<0.001	0.78 (0.68, 0.91)	<0.001
Robust	0.41 (0.36, 0.47)	<0.001	0.54 (0.49, 0.59)	<0.001	0.58 (0.52, 0.65)	<0.001
Part C. Mutually adjusted associations of intrinsic capacity and frailty index with all-cause mortality (*N* = 4,487)						
IC score, per 1-point increase	0.75 (0.74, 0.77)	<0.001	0.82 (0.81, 0.84)	<0.001	0.82 (0.81, 0.84)	<0.001
FI, per 0.1 increase	0.94 (0.90, 1.00)	0.033	1.02 (0.97, 1.08)	0.367	0.97 (0.91, 1.03)	0.296

Model 1 was unadjusted. Model 2 was adjusted for age and sex. Model 3 in Part A was additionally adjusted for smoking status, alcohol drinking status, residence, education level, marital status, economic status, self-rated health, self-rated quality of life, hypertension, diabetes, stroke, and heart disease. Model 3 in Parts B and C was additionally adjusted for smoking status, alcohol drinking status, residence, education level, marital status, economic status, and self-rated quality of life. FI categories were coded with frail as the reference group.

### Joint associations of intrinsic capacity and frailty index

Joint associations of IC and FI with incident disability and all-cause mortality are presented in Table 5. Using the higher IC + lower FI group as the reference, all three remaining joint categories exhibited higher risks of incident disability, with the greatest risk observed in the lower IC + higher FI group (OR = 4.31, 95% CI: 2.65–7.04). A similar gradient was observed for all-cause mortality, with the lower IC + higher FI group demonstrating the highest mortality risk (HR = 2.66, 95% CI: 2.32–3.04). Interaction analyses between IC and FI did not identify significant multiplicative or additive interactions for either incident disability or all-cause mortality (Supplementary Table 7). Kaplan–Meier curves for all-cause mortality according to joint IC-FI categories are presented in Supplementary Figure 3 (Figure 1). Kaplan–Meier analyses demonstrated significant differences in survival distributions across the four IC-FI groups (log-rank *p* < 0.001). *Post hoc* pairwise comparisons with Bonferroni correction showed that participants with high IC and non-frailty had significantly better survival than most other groups (Supplementary Table 8).

**Table 5:** Joint associations of intrinsic capacity and frailty index with incident disability and all-cause mortality.

Variables	Model 1	Model 1	Model 2	Model 2	Model 3	Model 3
	OR/HR (95% CI)	*p* value	OR/HR (95% CI)	*p* value	OR/HR (95% CI)	*p* value
Part A. Incident disability (*N* = 2,394)						
Higher IC + Lower FI	Reference		Reference		Reference	
Higher IC + Higher FI	1.94 (1.48, 2.53)	<0.001	1.89 (1.43, 2.50)	<0.001	1.91 (1.42, 2.55)	<0.001
Lower IC + Lower FI	3.83 (2.36, 6.21)	<0.001	1.34 (0.79, 2.28)	0.273	1.80 (1.00, 3.23)	0.050
Lower IC + Higher FI	5.65 (3.79, 8.44)	<0.001	2.83 (1.83, 4.37)	<0.001	4.31 (2.65, 7.04)	<0.001
Part B. All-cause mortality (*N* = 4,487)						
Higher IC + Lower FI	Reference		Reference		Reference	
Higher IC + Higher FI	1.30 (1.12, 1.51)	<0.001	1.30 (1.12, 1.51)	<0.001	1.28 (1.09, 1.49)	0.002
Lower IC + Lower FI	4.40 (3.84, 5.04)	<0.001	2.30 (1.98, 2.67)	<0.001	2.06 (1.75, 2.42)	<0.001
Lower IC + Higher FI	5.30 (4.76, 5.89)	<0.001	3.11 (2.75, 3.50)	<0.001	2.66 (2.32, 3.04)	<0.001

IC, intrinsic capacity; FI, frailty index; OR, odds ratio; CI, confidence interval; HR, hazard ratio. Incident disability was analyzed using logistic regression among participants without disability at baseline in 2014. All-cause mortality was analyzed using Cox proportional hazards models. Model 1 was unadjusted. Model 2 was adjusted for age and sex. Model 3 was additionally adjusted for smoking status, alcohol drinking status, residence, education level, marital status, economic status, and self-rated quality of life.

**Figure 1:**
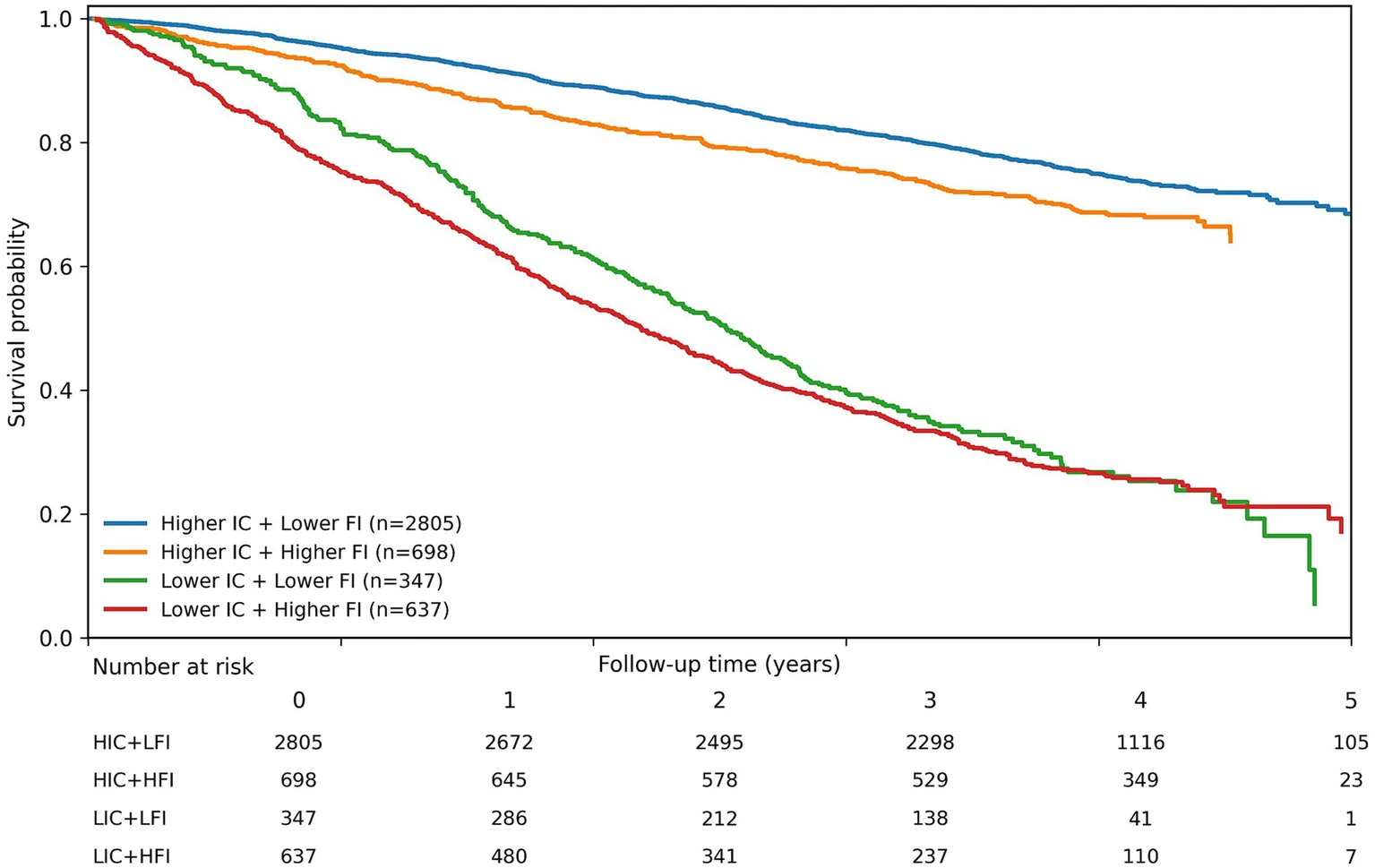
Kaplan–Meier curves for all-cause mortality according to joint categories of intrinsic capacity and frailty index.

### Sensitivity and incremental predictive analyses

Sensitivity analyses yielded results that were largely consistent with the primary findings. Restricting analyses to participants aged ≥65 years, excluding participants with unknown chronic disease history, and excluding deaths occurring within the first year of follow-up in mortality analyses did not materially alter the associations of IC and FI with incident disability or all-cause mortality (Supplementary Tables 9–11). Multinomial logistic regression analyses considering death as a separate outcome yielded results comparable to the primary analyses (Supplementary Table 12). Excluding self-rated health and self-rated quality of life from the adjustment models did not substantially change the observed associations, suggesting that the findings were robust to potential overadjustment (Supplementary Table 13). Multiple imputation analyses for missing covariates and modified Poisson regression with robust variance estimation for incident disability also produced results consistent with the primary analyses (Supplementary Tables 14–15).

## Discussion

In this prospective cohort study of older Chinese adults, lower IC and higher frailty, as assessed by the FI, were each independently associated with greater risks of incident disability and all-cause mortality. After mutual adjustment, both IC and FI remained significantly associated with incident disability, whereas only IC retained a significant association with all-cause mortality. Participants with concomitantly lower IC and higher FI consistently exhibited the highest risks of both outcomes.

Previous studies have linked lower IC and greater frailty to adverse functional outcomes in older adults, including disability and functional decline (9, 21–23). In the present study, each 1-point increase in IC was associated with a 21% lower odds of incident disability, whereas each 0.1-point increase in FI was associated with a 93% higher odds. Notably, both associations remained significant after mutual adjustment, although the magnitude of the effect estimates was attenuated. These findings suggest that IC and FI overlap to some degree but do not fully capture the same dimension of vulnerability relevant to functional decline. This interpretation is further supported by the domain-specific analyses, in which locomotion, vitality, sensory function, and cognition remained significantly associated with incident disability after full adjustment.

A different pattern emerged for all-cause mortality. Each 1-point increase in IC was associated with a 16% lower risk of death, whereas each 0.1-point increase in FI was associated with a 25% higher risk when the two indicators were evaluated separately. However, after mutual adjustment, only IC remained significantly associated with all-cause mortality. This attenuation of the FI association may reflect the conceptual and empirical overlap between the two constructs. FI summarizes the accumulation of multidimensional health deficits, many of which may also be manifested as reductions in physical or mental capacity, whereas IC more directly reflects preserved functional reserve. Consequently, part of the mortality-related information captured by FI may overlap with that represented by IC, leaving relatively limited FI-specific information after mutual adjustment. Conversely, the persistence of the IC association suggests that preserved capacity may capture aspects of health relevant to survival that are not fully represented by deficit accumulation alone. Nevertheless, this pattern should not be interpreted as demonstrating the superiority of IC over FI, because differences in construct composition, measurement characteristics, and residual confounding may also have contributed to the observed attenuation. Taken together, these findings suggest that the mortality-related information captured by FI may substantially overlap with that captured by IC in this cohort. Comparative evidence further suggests that the relative contributions of IC and frailty measures may vary according to outcome. In the Sydney Memory and Aging Study, IC explained additional variance beyond frailty measures (FI and Fried frailty phenotype) for incident dementia, whereas its contribution beyond FI for mortality was limited (24). By contrast, the present study found that IC retained an independent association with all-cause mortality after accounting for FI. Differences in population characteristics, follow-up duration, and the operationalization of IC and FI may partly account for these between-study discrepancies.

The joint analyses provide additional perspective. Participants with lower IC and higher FI consistently had the highest risks of both incident disability and all-cause mortality, supporting the value of jointly considering these constructs when characterizing health vulnerability in older adults. This finding is consistent with previous evidence indicating that co-occurring low IC and frailty are associated with poorer self-rated health than either condition alone (25). The observed pattern is also biologically plausible, as IC-frailty phenotypes have been shown to follow a gradient of subclinical inflammation, with the most unfavorable phenotype characterized by lower IC and frailty exhibiting the highest inflammatory burden (26). Taken together, these findings suggest that the joint consideration of IC and FI may provide a more comprehensive characterization of vulnerability than either construct considered in isolation, particularly for functional outcomes.

These findings may be interpreted in light of the distinct conceptual foundations of IC and FI. IC reflects preserved physical and mental capacities, whereas FI captures the accumulation of multidimensional deficits. For disability, both diminished reserve and increased deficit burden are likely to be important, which may explain why IC and FI remained independently associated after mutual adjustment. For mortality, preserved overall capacity may be more closely related to survival once the shared information between the two measures is accounted for. From a practical perspective, IC and FI may provide complementary information regarding health vulnerability in older adults. Rather than requiring comprehensive assessment of both measures at every clinical encounter, a staged approach may be more feasible, with IC potentially serving as an initial assessment of preserved capacity and FI providing complementary information on accumulated deficits when greater vulnerability is suspected or sufficient clinical data are available. Joint interpretation may be particularly useful for identifying individuals with concordantly unfavorable profiles who may benefit from closer functional monitoring and more comprehensive geriatric assessment. However, further implementation studies are needed before such an approach can be incorporated into routine clinical pathways or formal guidelines.

This study has several strengths. It was based on a large nationwide cohort of older Chinese adults and simultaneously examined incident disability and all-cause mortality. It also evaluated IC and FI within the same analytical framework, including separate, mutually adjusted, and joint analyses, thereby allowing their independent and combined associations with adverse outcomes to be examined. Domain-specific analyses and a series of sensitivity analyses addressing population selection, missing data, covariate adjustment, and alternative modeling approaches further supported the robustness of the findings.

Several limitations should also be acknowledged. First, this was an observational study, and causal relationships cannot be established; residual or unmeasured confounding may remain despite multivariable adjustment. Second, some components used to construct IC and FI were based on self-reported information and may therefore be subject to measurement error. Third, self-rated health and self-rated quality of life may conceptually overlap with IC and FI or lie partly on pathways linking these constructs to subsequent outcomes, raising the possibility of overadjustment. However, sensitivity analyses excluding these variables yielded broadly consistent findings. Fourth, the exact timing of incident disability was unavailable, precluding conventional time-to-event competing-risk analyses; we therefore addressed death before follow-up assessment using multinomial outcome modeling as a sensitivity analysis. Fifth, participants included in the final analytical samples differed from those excluded because of missing data, which may have introduced selection bias. Although multiple imputation of missing covariates yielded broadly consistent results, potential bias related to missing exposure or outcome information could not be fully addressed. Therefore, the findings should be interpreted with some caution, particularly for older adults with poorer health status or incomplete follow-up. Finally, because the findings were derived from older Chinese adults, their generalizability to populations with different demographic, socioeconomic, and healthcare contexts should be considered with caution.

## Conclusion

In conclusion, both lower IC and higher FI were associated with increased risks of incident disability and all-cause mortality among older Chinese adults. For incident disability, IC and FI retained independent associations after mutual adjustment, and their combined assessment revealed heterogeneous risk profiles. For all-cause mortality, IC remained independently associated with mortality, whereas the association between FI and mortality was attenuated after adjustment for IC, suggesting substantial overlap in the mortality-related information captured by the two constructs. These findings suggest that IC and FI provide complementary information regarding functional vulnerability and adverse outcomes, whereas IC showed a stronger independent association with survival in later life.

## Data Availability

Publicly available datasets were analyzed in this study. This data can be found here: https://opendata.pku.edu.cn/dataverse/CHADS.
