# Supplementary material for: Independent and joint associations of intrinsic capacity and frailty index with incident disability and all-cause mortality among older Chinese adults
Source: Front Public Health. 2026 Sep 16;14:1859572. doi: 10.3389/fpubh.2026.1859572 (PMC13623747; doi:10.3389/fpubh.2026.1859572)
Supplement: Supplementary file 1 [file Supplementary_file_1.docx]

**Independent and** **joint associations of intrinsic capacity and frailty index with incident disability and all-cause mortality among older Chinese adults**

Bing Feng^1^**^†^**, Ji Wang^1^**^†^**, Yaowen Li^1^, Liangliang Ma^2^, Yue Yin^3^, Juan Xu^4*^, Yuhan Dong^1*^

^1^ Department of Traditional Chinese Medicine Rehabilitation The 966th Hospital of The PLA Joint Logistic Support Force of China, Dandong, Liaoning, 118000, China

^2^ Outpatient Department of the 15th Veteran Cadre Sanatorium of Beijing Garrison Command, Beijing, 100093, China

^3^ Outpatient Department, Liaoning Provincial Military District Dandong First Retired Cadres Sanatorium, Dandong, Liaoning, 118002, China

^4^ Department of General Surgery, Affiliated Xiaoshan Hospital, Hangzhou Normal University, Hangzhou, 311200, China

*** Correspondence:**

Juan Xu ([sophia2932@163.com](mailto:sophia2932@163.com))

Yuhan Dong (dyh1994727@163.com)

**† Ji Wang and Bing Feng contributed equally to this work and share first authorship.**

**Supplementary Methods**

**Assessment of interactions between IC and FI**

Interaction analyses between IC and FI were conducted on both multiplicative and additive scales. Multiplicative interactions were assessed by including an IC×FI product term in multivariable regression models. For additive interactions, the relative excess risk due to interaction (RERI), attributable proportion due to interaction (AP), and synergy index (S) were calculated using the group with higher IC and non-frailty as the reference category. A RERI or AP value of zero and a synergy index of one indicated no additive interaction. Confidence intervals were estimated using the delta method.

**Restricted cubic spline analyses**
Restricted cubic spline (RCS) models were used to examine the dose–response relationships of continuous intrinsic capacity (IC) and frailty index (FI) with incident disability and all-cause mortality. Five knots were placed at the five knots at the 5th, 27.5th, 50th, 72.5th, and 95th percentiles of the distribution of each exposure. The median values of IC (7 points) and FI (0.17) were used as the reference values, corresponding to an odds ratio or hazard ratio of 1.00. The RCS models were adjusted using the same covariate sets as the corresponding fully adjusted Model 3 analyses. P values for the overall association were obtained by jointly testing all spline terms, whereas P values for nonlinearity were obtained by jointly testing the nonlinear spline terms. A P value for nonlinearity <0.05 was considered evidence of a nonlinear association.

**Sensitivity analyses**

Sensitivity analyses were performed to evaluate the robustness of the main findings. First, we repeated the analyses under alternative population definitions and outcome specifications. These analyses included restricting participants to those aged ≥65 years, excluding participants with unknown chronic disease history, excluding deaths occurring within the first year of follow-up in mortality analyses, and applying multinomial logistic regression for incident disability analyses to account for the competing occurrence of death before follow-up assessment. Second, to assess potential overadjustment, analyses were repeated after removing self-rated health and self-rated quality of life from the adjustment models. Third, multiple imputation by chained equations (MICE) was conducted to evaluate the potential impact of missing covariate data. Twenty imputed datasets were generated, with imputation restricted to covariates only; IC, FI, outcome variables, and survival time were not imputed. Regression models were repeated across imputed datasets and pooled using Rubin’s rules. Finally, modified Poisson regression with robust variance estimation was performed as an alternative approach for incident disability analyses to estimate risk ratios (RRs) and 95% confidence intervals (CIs), given that the incidence of incident disability exceeded the threshold for rare outcomes.

**Supplementary Table 1. Definitions and coding of intrinsic capacity domains and frailty index items**

**Panel A. Intrinsic capacity domains**

| **Domain** | **Indicator** | **Question / assessment** | **Coding** |
| --- | --- | --- | --- |
| Locomotion | Walking ability | Can you walk continuously for 1 kilometer at a time by yourself? | Yes, independently = 2; Yes, but need some help = 1; No, cannot = 0 |
| Sensory | Hearing difficulty | Do you have any difficulty with your hearing? | No = 1; Yes, left ear or right ear = 0.5; Yes, both ears = 0 |
| Sensory | Vision | Can the interviewee see a break in the circle on the cardboard sheet when lit by a flashlight and distinguish where the break is located? | Can see and distinguish = 1; Can see only = 0.5; Cannot see = 0; Blind = 0 |
| Vitality | Lifting ability | Can you lift a weight of 5 kg, such as a heavy bag of groceries? | Yes, independently = 2; Yes, but need some help = 1; No, cannot = 0 |
| Psychological | Psychological resilience | Five questions were used: (1) Do you always look on the bright side of things? (2) Can you make your own decisions concerning your personal affairs? (3) Do you often feel fearful or anxious? (4) Do you often feel lonely and isolated? (5) Do you feel the older you get, the more useless you are? | Questions 1-2: Always = 5, Often = 4, Sometimes = 3, Seldom = 2, Never = 1; Questions 3-5: Always = 1, Often = 2, Sometimes = 3, Seldom = 4, Never = 5. Total score ranges from 5 to 25. Tertiles were used: <=16 = 0; 17-19 = 1; >=20 = 2 |
| Cognition | MMSE | Chinese version of the Mini-Mental State Examination (MMSE) | >=24 = 2; 18-23 = 1; <18 = 0 |

**Panel B. Frailty index items**

| **No.** | **Domain** | **Variable** | **Coding** |
| --- | --- | --- | --- |
| **COMORBIDITIES** | | | |
| 1 | Comorbidities | Suffering from hypertension | Yes = 1; Suspect = 0.5; No = 0 |
| 2 | Comorbidities | Suffering from diabetes | Yes = 1; Suspect = 0.5; No = 0 |
| 3 | Comorbidities | Suffering from heart disease | Yes = 1; Suspect = 0.5; No = 0 |
| 4 | Comorbidities | Suffering from stroke; cerebrovascular disease | Yes = 1; Suspect = 0.5; No = 0 |
| 5 | Comorbidities | Suffering from bronchitis; emphysema; asthma; pneumonia | Yes = 1; Suspect = 0.5; No = 0 |
| 6 | Comorbidities | Suffering from tuberculosis | Yes = 1; Suspect = 0.5; No = 0 |
| 7 | Comorbidities | Suffering from cataract | Yes = 1; Suspect = 0.5; No = 0 |
| 8 | Comorbidities | Suffering from cancer | Yes = 1; Suspect = 0.5; No = 0 |
| 9 | Comorbidities | Suffering from gastric or duodenal ulcer | Yes = 1; Suspect = 0.5; No = 0 |
| 10 | Comorbidities | Suffering from bedsore | Yes = 1; Suspect = 0.5; No = 0 |
| 11 | Comorbidities | Suffering from arthritis | Yes = 1; Suspect = 0.5; No = 0 |
| 12 | Comorbidities | Suffering from chronic nephritis | Yes = 1; Suspect = 0.5; No = 0 |
| 13 | Comorbidities | Suffering from hepatitis | Yes = 1; Suspect = 0.5; No = 0 |
| **SYMPTOMS** | | | |
| 14 | Symptoms | Have a toothache | Yes = 1; No = 0 |
| 15 | Symptoms | Have pain in the jaw joint or in front of the ear or across your face or cheek | Yes = 1; No = 0 |
| **SLEEP** | | | |
| 16 | Sleep | Sleep quality | Very bad, Bad = 1; So so = 0.5; Good, Very good = 0 |
| 17 | Sleep | Sleep duration | ≤4, ≥10 hours = 1; 5-6, 9-10 hours = 0.5; 7-8 hours = 0 |
| **HEALTH STATUS** | | | |
| 18 | Health Status | Self-rated health at present | Very bad, Bad = 1; So so = 0.5; Good, Very good = 0 |
| 19 | Health Status | Self-rated health compared with before | Worse = 1, Slightly Worse = 0.5; Same, Better = 0 |
| 20 | Health Status | Number of times suffering from serious illness in the past two years | 0 = 0; ≥1 = 1 |
| **PHYSICAL PERFORMANCE** | | | |
| 21 | Physical Performance | Housework at present | Almost every day = 0; Not daily, but at least once a week = 0.25; Not weekly, but at least once a month = 0.5; Not monthly, but sometimes = 0.75; Never = 1 |
| 22 | Physical Performance | Hand behind neck | Right hand = 0.5; Left hand = 0.5; Both hands = 0; Neither hand = 1 |
| 23 | Physical Performance | Hand behind lower back | Right hand = 0.5; Left hand = 0.5; Both hands = 0; Neither hand = 1 |
| 24 | Physical Performance | Raise arms upright | Right hand = 0.5; Left hand = 0.5; Both hands = 0; Neither hand = 1 |
| 25 | Physical Performance | Able to stand up from sitting | Yes, without using hands = 0; Yes, using hands = 0.5; No = 1 |
| 26 | Physical Performance | Able to pick up a book from the floor | Yes, standing = 0; Yes, sitting = 0.5; No = 1 |
| 27 | Physical Performance | Able to continuously crouch and stand up three times | Yes, independently = 0; Yes, but need some help = 0.5; No = 1 |
| 28 | Physical Performance | Able to use chopsticks to eat | Yes = 0; No = 1 |
| 29 | Physical Performance | Able to visit neighbors by yourself | Yes, independently = 0; Yes, but need some help = 0.5; No = 1 |
| 30 | Physical Performance | Number of steps used to turn around a 360 degree turn without help | ≤4 = 0; >4 = 0.5; Cannot turn around = 1 |
| 31 | Physical Performance | BMI | <18.5; >28 = 1; 24-28 = 0.5; 18.5-24 = 0 |
| 32 | Physical Performance | Cardiac rhythm | Regular = 0; Irregular = 1 |
| 33 | Physical Performance | Have false teeth | Yes = 1; No = 0 |
| **DEPRESSION** | | | |
| 34 | Depression | Keep my belongings neat and clean | Always = 0; Often = 0.25; Sometimes = 0.5; Seldom = 0.75; Never = 1 |
| 35 | Depression | Feel as happy as when you were younger | Same = 0; Often = 0.25; Sometimes = 0.5; Seldom = 0.75; Never = 1 |
| 36 | Depression | Felt sad; blue; or depressed | Yes = 1; No = 0 |
| 37 | Depression | Lost interest in most things | Yes = 1; No = 0 |

*Abbreviations: CLHLS, Chinese Longitudinal Healthy Longevity Survey; MMSE, Mini-Mental State Examination.*

**Supplementary Table 2. Comparison of baseline characteristics between participants included in and excluded from the joint IC-FI Model 3 for incident disability in the total study population**

| Characteristic | Included (N = 2394) | Excluded (N = 4774) | P value |
| --- | --- | --- | --- |
| Age, years | 80.42 ± 8.74 | 87.93 ± 10.62 | <0.001 |
| Sex |  |  | <0.001 |
| female | 1183 (49.4%) | 2688 (56.3%) |  |
| male | 1211 (50.6%) | 2086 (43.7%) |  |
| Smoking status |  |  | <0.001 |
| Never | 1623 (67.8%) | 3304 (71.6%) |  |
| Current | 468 (19.5%) | 697 (15.1%) |  |
| Former | 303 (12.7%) | 615 (13.3%) |  |
| Alcohol drinking status |  |  | <0.001 |
| Never | 1718 (71.8%) | 3526 (76.9%) |  |
| Current | 445 (18.6%) | 616 (13.4%) |  |
| Former | 231 (9.6%) | 446 (9.7%) |  |
| Residence |  |  | <0.001 |
| Rural | 1351 (56.4%) | 2616 (54.8%) |  |
| Town | 771 (32.2%) | 1443 (30.2%) |  |
| City | 272 (11.4%) | 715 (15.0%) |  |
| Education level, years |  |  | <0.001 |
| <=5 | 1824 (76.2%) | 3884 (82.4%) |  |
| >5 | 570 (23.8%) | 829 (17.6%) |  |
| Marital status |  |  | <0.001 |
| Unmarried/divorced/widowed | 1136 (47.5%) | 3041 (66.6%) |  |
| Married | 1258 (52.5%) | 1527 (33.4%) |  |
| Economic status |  |  | <0.001 |
| Fair | 1754 (73.3%) | 3247 (71.6%) |  |
| Rich | 427 (17.8%) | 700 (15.4%) |  |
| Poor | 213 (8.9%) | 591 (13.0%) |  |
| Self-rated health |  |  | <0.001 |
| Good or very good | 1185 (49.5%) | 1724 (41.5%) |  |
| Fair | 937 (39.2%) | 1693 (40.7%) |  |
| Poor or very poor | 270 (11.3%) | 739 (17.8%) |  |
| Self-rated quality of life |  |  | <0.001 |
| Good or very good | 1615 (67.5%) | 2756 (66.3%) |  |
| Fair | 726 (30.3%) | 1210 (29.1%) |  |
| Poor or very poor | 53 (2.2%) | 193 (4.6%) |  |
| Hypertension |  |  | <0.001 |
| No | 1506 (63.0%) | 2839 (63.6%) |  |
| Yes | 840 (35.1%) | 1395 (31.3%) |  |
| Unknown | 46 (1.9%) | 227 (5.1%) |  |
| Diabetes |  |  | <0.001 |
| No | 2213 (92.5%) | 3892 (88.5%) |  |
| Yes | 125 (5.2%) | 257 (5.8%) |  |
| Unknown | 55 (2.3%) | 247 (5.6%) |  |
| Stroke |  |  | <0.001 |
| No | 2059 (86.1%) | 3568 (81.0%) |  |
| Yes | 287 (12.0%) | 604 (13.7%) |  |
| Unknown | 45 (1.9%) | 235 (5.3%) |  |
| Heart disease |  |  | <0.001 |
| No | 2176 (90.9%) | 3749 (85.2%) |  |
| Yes | 177 (7.4%) | 428 (9.7%) |  |
| Unknown | 41 (1.7%) | 221 (5.0%) |  |
| Intrinsic capacity score | 8.05 ± 2.00 | 5.40 ± 3.22 | <0.001 |
| Frailty index | 0.16 ± 0.08 | 0.20 ± 0.11 | <0.001 |

Included: N = 2394; Excluded: N = 4774. Continuous variables are presented as mean ± standard deviation, and categorical variables are presented as n (%). P values were calculated using Welch's t test for continuous variables and the chi-square test for categorical variables.

**Supplementary Table 3. Comparison of baseline characteristics between participants included in and excluded from the joint IC-FI Model 3 for all-cause mortality in the total study population**

| Characteristic | Included (N = 4487) | Excluded (N = 2681) | P value |
| --- | --- | --- | --- |
| Age, years | 84.47 ± 10.34 | 87.00 ± 10.95 | <0.001 |
| Sex |  |  | <0.001 |
| female | 2333 (52.0%) | 1538 (57.4%) |  |
| male | 2154 (48.0%) | 1143 (42.6%) |  |
| Smoking status |  |  | <0.001 |
| Never | 3110 (69.3%) | 1817 (72.0%) |  |
| Current | 761 (17.0%) | 404 (16.0%) |  |
| Former | 616 (13.7%) | 302 (12.0%) |  |
| Alcohol drinking status |  |  | <0.001 |
| Never | 3310 (73.8%) | 1934 (77.5%) |  |
| Current | 715 (15.9%) | 346 (13.9%) |  |
| Former | 462 (10.3%) | 215 (8.6%) |  |
| Residence |  |  | <0.001 |
| Rural | 2529 (56.4%) | 1438 (53.6%) |  |
| Town | 1459 (32.5%) | 755 (28.2%) |  |
| City | 499 (11.1%) | 488 (18.2%) |  |
| Education level, years |  |  | <0.001 |
| <=5 | 3618 (80.6%) | 2090 (79.8%) |  |
| >5 | 869 (19.4%) | 530 (20.2%) |  |
| Marital status |  |  | <0.001 |
| Unmarried/divorced/widowed | 2583 (57.6%) | 1594 (64.4%) |  |
| Married | 1904 (42.4%) | 881 (35.6%) |  |
| Economic status |  |  | <0.001 |
| Fair | 3255 (72.5%) | 1746 (71.4%) |  |
| Rich | 763 (17.0%) | 364 (14.9%) |  |
| Poor | 469 (10.5%) | 335 (13.7%) |  |
| Self-rated health |  |  | <0.001 |
| Good or very good | 2048 (45.8%) | 861 (41.6%) |  |
| Fair | 1767 (39.5%) | 863 (41.7%) |  |
| Poor or very poor | 661 (14.8%) | 348 (16.8%) |  |
| Self-rated quality of life |  |  | <0.001 |
| Good or very good | 2997 (66.8%) | 1374 (66.5%) |  |
| Fair | 1348 (30.0%) | 588 (28.5%) |  |
| Poor or very poor | 142 (3.2%) | 104 (5.0%) |  |
| Hypertension |  |  | <0.001 |
| No | 2865 (63.9%) | 1480 (62.4%) |  |
| Yes | 1478 (33.0%) | 757 (31.9%) |  |
| Unknown | 139 (3.1%) | 134 (5.7%) |  |
| Diabetes |  |  | <0.001 |
| No | 4087 (91.1%) | 2018 (87.6%) |  |
| Yes | 242 (5.4%) | 140 (6.1%) |  |
| Unknown | 156 (3.5%) | 146 (6.3%) |  |
| Stroke |  |  | <0.001 |
| No | 3786 (84.5%) | 1841 (79.5%) |  |
| Yes | 554 (12.4%) | 337 (14.6%) |  |
| Unknown | 142 (3.2%) | 138 (6.0%) |  |
| Heart disease |  |  | <0.001 |
| No | 3996 (89.1%) | 1929 (83.6%) |  |
| Yes | 351 (7.8%) | 254 (11.0%) |  |
| Unknown | 137 (3.1%) | 125 (5.4%) |  |
| Intrinsic capacity score | 6.79 ± 2.86 | 5.46 ± 3.43 | <0.001 |
| Frailty index | 0.18 ± 0.10 | 0.21 ± 0.12 | <0.001 |

Included: N = 4487; Excluded: N = 2681. Continuous variables are presented as mean ± standard deviation, and categorical variables are presented as n (%). P values were calculated using Welch's t test for continuous variables and the chi-square test for categorical variables.

**Supplementary Table 4.** **Baseline characteristics of the participants according to dichotomized intrinsic capacity and frailty index categories**

| Variable | Overall (N=6,178) | Higher IC + Lower FI (n=3,585) | Higher IC + Higher FI (n=929) | Lower IC + Lower FI (n=533) | Lower IC + Higher FI (n=1,131) | P value |
| --- | --- | --- | --- | --- | --- | --- |
| Age, years | 85.3 ± 10.6 | 81.8 ± 9.3 | 83.0 ± 9.0 | 95.6 ± 7.9 | 93.2 ± 9.3 | <0.001 |
| Sex |  |  |  |  |  | <0.001 |
| Male | 2844 (46.0) | 1935 (54.0) | 428 (46.1) | 132 (24.8) | 349 (30.9) |  |
| Female | 3334 (54.0) | 1650 (46.0) | 501 (53.9) | 401 (75.2) | 782 (69.1) |  |
| Education level, years |  |  |  |  |  | <0.001 |
| <=5 | 4941 (80.6) | 2700 (75.8) | 721 (77.9) | 500 (95.1) | 1020 (91.1) |  |
| >5 | 1192 (19.4) | 861 (24.2) | 205 (22.1) | 26 (4.9) | 100 (8.9) |  |
| Marital status |  |  |  |  |  | <0.001 |
| Married | 2446 (40.0) | 1760 (49.6) | 399 (43.4) | 63 (12.0) | 224 (20.0) |  |
| Unmarried/divorced/widowed | 3672 (60.0) | 1790 (50.4) | 521 (56.6) | 463 (88.0) | 898 (80.0) |  |
| Smoking status |  |  |  |  |  | <0.001 |
| Never | 4293 (69.8) | 2336 (65.5) | 607 (65.6) | 446 (84.0) | 904 (80.3) |  |
| Former | 826 (13.4) | 455 (12.7) | 176 (19.0) | 47 (8.9) | 148 (13.1) |  |
| Current | 1033 (16.8) | 778 (21.8) | 143 (15.4) | 38 (7.2) | 74 (6.6) |  |
| Alcohol drinking status |  |  |  |  |  | <0.001 |
| Never | 4601 (75.0) | 2549 (71.7) | 658 (71.6) | 463 (87.7) | 931 (82.6) |  |
| Former | 607 (9.9) | 307 (8.6) | 148 (16.1) | 27 (5.1) | 125 (11.1) |  |
| Current | 923 (15.1) | 701 (19.7) | 113 (12.3) | 38 (7.2) | 71 (6.3) |  |
| Residence |  |  |  |  |  | <0.001 |
| Rural | 3372 (54.6) | 1992 (55.6) | 402 (43.3) | 368 (69.0) | 610 (53.9) |  |
| Town | 1962 (31.8) | 1147 (32.0) | 335 (36.1) | 127 (23.8) | 353 (31.2) |  |
| City | 844 (13.7) | 446 (12.4) | 192 (20.7) | 38 (7.1) | 168 (14.9) |  |
| Economic status |  |  |  |  |  | <0.001 |
| Poor | 684 (11.2) | 273 (7.6) | 131 (14.1) | 65 (12.5) | 215 (19.8) |  |
| Average | 4415 (72.4) | 2614 (73.2) | 649 (70.1) | 394 (75.6) | 758 (69.9) |  |
| Rich | 1002 (16.4) | 682 (19.1) | 146 (15.8) | 62 (11.9) | 112 (10.3) |  |
| Self-rated health |  |  |  |  |  | <0.001 |
| Poor/very poor | 868 (14.9) | 188 (5.3) | 318 (34.5) | 44 (9.2) | 318 (36.4) |  |
| Fair | 2340 (40.1) | 1323 (37.1) | 424 (46.0) | 213 (44.5) | 380 (43.5) |  |
| Good/very good | 2634 (45.1) | 2057 (57.7) | 180 (19.5) | 222 (46.3) | 175 (20.0) |  |
| Self-rated quality of life |  |  |  |  |  | <0.001 |
| Poor/very poor | 204 (3.5) | 46 (1.3) | 51 (5.5) | 22 (4.6) | 85 (9.7) |  |
| Fair | 1713 (29.3) | 941 (26.4) | 314 (34.1) | 130 (27.1) | 328 (37.4) |  |
| Good/very good | 3931 (67.2) | 2583 (72.4) | 557 (60.4) | 327 (68.3) | 464 (52.9) |  |
| Hypertension |  |  |  |  |  | <0.001 |
| No | 3949 (64.0) | 2498 (69.8) | 353 (38.0) | 447 (84.2) | 651 (57.6) |  |
| Unknown | 238 (3.9) | 51 (1.4) | 61 (6.6) | 8 (1.5) | 118 (10.4) |  |
| Yes | 1984 (32.2) | 1032 (28.8) | 515 (55.4) | 76 (14.3) | 361 (31.9) |  |
| Diabetes |  |  |  |  |  | <0.001 |
| No | 5573 (90.3) | 3422 (95.6) | 705 (76.0) | 522 (97.9) | 924 (81.7) |  |
| Unknown | 262 (4.2) | 34 (0.9) | 82 (8.8) | 6 (1.1) | 140 (12.4) |  |
| Yes | 338 (5.5) | 125 (3.5) | 141 (15.2) | 5 (0.9) | 67 (5.9) |  |
| Stroke |  |  |  |  |  | <0.001 |
| No | 5139 (83.3) | 3277 (91.5) | 528 (57.1) | 506 (94.9) | 828 (73.5) |  |
| Unknown | 244 (4.0) | 29 (0.8) | 86 (9.3) | 6 (1.1) | 123 (10.9) |  |
| Yes | 783 (12.7) | 277 (7.7) | 310 (33.5) | 21 (3.9) | 175 (15.5) |  |
| Heart disease |  |  |  |  |  | <0.001 |
| No | 5407 (87.6) | 3418 (95.3) | 641 (69.2) | 521 (97.7) | 827 (73.3) |  |
| Unknown | 231 (3.7) | 19 (0.5) | 88 (9.5) | 2 (0.4) | 122 (10.8) |  |
| Yes | 535 (8.7) | 148 (4.1) | 197 (21.3) | 10 (1.9) | 180 (15.9) |  |

*Abbreviations: FI, frailty index; IC, intrinsic capacity.*

P values were calculated using one-way analysis of variance for continuous variables and the chi-square test for categorical variables. Percentages were calculated among participants with available data for each variable, and thus may not sum to 100% because of missing values.

**Supplementary Table 5. Associations of the five intrinsic capacity domains with incident disability from 2014 to 2018**

| Domain | N | Events | Model 1 OR (95% CI) | P value | Model 2 OR (95% CI) | P value | Model 3 OR (95% CI) | P value |
| --- | --- | --- | --- | --- | --- | --- | --- | --- |
| Locomotion | 2555 | 432 | 0.35 (0.31, 0.41) | <0.001 | 0.49 (0.42, 0.57) | <0.001 | **0.51 (0.43, 0.60)** | <0.001 |
| Vitality | 2557 | 432 | 0.40 (0.35, 0.46) | <0.001 | 0.56 (0.48, 0.66) | <0.001 | **0.59 (0.50, 0.70)** | <0.001 |
| Sensory | 2522 | 429 | 0.40 (0.34, 0.47) | <0.001 | 0.58 (0.48, 0.69) | <0.001 | **0.58 (0.48, 0.69)** | <0.001 |
| Psychological | 2555 | 430 | 0.85 (0.75, 0.98) | 0.021 | 1.02 (0.88, 1.18) | 0.819 | 1.04 (0.88, 1.22) | 0.646 |
| Cognition | 2439 | 412 | 0.44 (0.37, 0.53) | <0.001 | 0.69 (0.56, 0.85) | <0.001 | **0.67 (0.54, 0.82)** | <0.001 |

Model 1 was unadjusted. Model 2 was adjusted for age and sex. Model 3 was adjusted for age, sex, smoking status, alcohol drinking status, residence, education level, marital status, economic status, self-rated health, self-rated quality of life, and history of hypertension, diabetes, stroke, and heart disease. Sample size varied across domains because complete-case analyses were performed for each domain-specific model.

**Supplementary Table 6. Associations of the five intrinsic capacity domains with all-cause mortality from 2014 to 2018**

| Domain | N | Deaths | Model 1 HR (95% CI) | P value | Model 2 HR (95% CI) | P value | Model 3 HR (95% CI) | P value |
| --- | --- | --- | --- | --- | --- | --- | --- | --- |
| Locomotion | 4778 | 1725 | 0.43 (0.41, 0.45) | <0.001 | 0.56 (0.53, 0.59) | <0.001 | **0.61 (0.58, 0.66)** | <0.001 |
| Vitality | 4782 | 1726 | 0.44 (0.42, 0.46) | <0.001 | 0.57 (0.54, 0.60) | <0.001 | **0.63 (0.59, 0.68)** | <0.001 |
| Sensory | 4709 | 1697 | 0.44 (0.42, 0.47) | <0.001 | 0.67 (0.63, 0.71) | <0.001 | **0.75 (0.70, 0.81)** | <0.001 |
| Psychological | 4778 | 1722 | 0.60 (0.57, 0.63) | <0.001 | 0.72 (0.68, 0.76) | <0.001 | **0.82 (0.77, 0.88)** | <0.001 |
| Cognition | 4575 | 1663 | 0.44 (0.42, 0.46) | <0.001 | 0.61 (0.57, 0.64) | <0.001 | **0.67 (0.63, 0.72)** | <0.001 |

Model 1 was unadjusted. Model 2 was adjusted for age and sex. Model 3 was adjusted for age, sex, education level, marital status, smoking status, alcohol drinking status, residence, economic status, self-rated health, self-rated quality of life, and history of hypertension, diabetes, stroke, and heart disease. Sample size varied across domains because complete-case analyses were performed for each domain-specific model.

**Supplementary Table 7. Additive and multiplicative interactions between intrinsic capacity and frailty for incident disability and all-cause mortality**

| Outcome | Multiplicative interaction (IC×FI) OR/HR (95% CI) | P for interaction | RERI (95% CI) | AP (95% CI) | Synergy index (S) (95% CI) |
| --- | --- | --- | --- | --- | --- |
| Incident disability | 0.763 (0.396–1.472) | 0.420 | 0.892 (−1.929, 3.712) | 0.158 (−0.298, 0.614) | 1.237 (0.406, 2.068) |
| All-cause mortality | 0.925 (0.755–1.132) | 0.447 | 0.587 (−0.085, 1.259) | 0.110 (−0.012, 0.232) | 1.156 (0.958, 1.355) |

Notes: IC, intrinsic capacity; FI, frailty index; RERI, relative excess risk due to interaction; AP, attributable proportion due to interaction. Reference group for joint analyses was high IC + non-frail.

**Supplementary Table 8. Post hoc pairwise comparisons of survival distributions among IC-FI joint groups**

| Comparison | Log-rank χ² | P value | Bonferroni-adjusted P value |
| --- | --- | --- | --- |
| High IC + Non-frail vs High IC + Frail | 11.54 | 0.0007 | 0.0041 |
| High IC + Non-frail vs Low IC + Non-frail | 578.22 | <0.001 | <0.001 |
| High IC + Non-frail vs Low IC + Frail | 1173.83 | <0.001 | <0.001 |
| High IC + Frail vs Low IC + Non-frail | 211.67 | <0.001 | <0.001 |
| High IC + Frail vs Low IC + Frail | 361.54 | <0.001 | <0.001 |
| Low IC + Non-frail vs Low IC + Frail | 6.83 | 0.0090 | 0.0537 |

Note: Post hoc pairwise comparisons were performed after the overall log-rank test. P values were adjusted for multiple comparisons using the Bonferroni method.

## Supplementary Table 9. Sensitivity analysis restricted to participants aged ≥65 years (Model 3)

| **Variables** | **Effect estimate (95% CI)** | **P value** |
| --- | --- | --- |
| **Incident disability** |  |  |
| IC per 1-point increase (IC-only model) | 0.79 (0.74–0.84) | <0.001 |
| FI per 0.1 increase (FI-only model) | 1.93 (1.67–2.22) | <0.001 |
| IC per 1-point increase (mutually adjusted model) | 0.84 (0.79–0.90) | <0.001 |
| FI per 0.1 increase (mutually adjusted model) | 1.69 (1.43–2.00) | <0.001 |
| Higher IC + Lower FI | Reference | — |
| Higher IC + Higher FI | 1.83 (1.38–2.42) | <0.001 |
| Lower IC + Lower FI | 1.81 (1.05–3.12) | 0.034 |
| Lower IC + Higher FI | 4.03 (2.49–6.50) | <0.001 |
| **All-cause mortality** |  |  |
| IC per 1-point increase (IC-only model) | 0.84 (0.82–0.85) | <0.001 |
| FI per 0.1 increase (FI-only model) | 1.25 (1.19–1.31) | <0.001 |
| IC per 1-point increase (mutually adjusted model) | 0.82 (0.81–0.84) | <0.001 |
| FI per 0.1 increase (mutually adjusted model) | 0.97 (0.91–1.03) | 0.296 |
| Higher IC + Lower FI | Reference | — |
| Higher IC + Higher FI | 1.33 (1.15–1.53) | <0.001 |
| Lower IC + Lower FI | 2.05 (1.76–2.39) | <0.001 |
| Lower IC + Higher FI | 2.66 (2.34–3.03) | <0.001 |

## Supplementary Table 10. Sensitivity analysis excluding participants with unknown chronic disease history (Model 3)

| **Variables** | **Effect estimate (95% CI)** | **P value** |
| --- | --- | --- |
| **Incident disability** |  |  |
| IC per 1-point increase (IC-only model) | 0.79 (0.74–0.85) | <0.001 |
| FI per 0.1 increase (FI-only model) | 2.02 (1.73–2.36) | <0.001 |
| IC per 1-point increase (mutually adjusted model) | 0.84 (0.78–0.90) | <0.001 |
| FI per 0.1 increase (mutually adjusted model) | 1.72 (1.45–2.05) | <0.001 |
| Higher IC + Lower FI | Reference | — |
| Higher IC + Higher FI | 2.01 (1.50–2.69) | <0.001 |
| Lower IC + Lower FI | 1.79 (1.03–3.10) | 0.039 |
| Lower IC + Higher FI | 3.64 (2.16–6.13) | <0.001 |
| **All-cause mortality** |  |  |
| IC per 1-point increase (IC-only model) | 0.84 (0.82–0.85) | <0.001 |
| FI per 0.1 increase (FI-only model) | 1.27 (1.20–1.35) | <0.001 |
| IC per 1-point increase (mutually adjusted model) | 0.82 (0.81–0.84) | <0.001 |
| FI per 0.1 increase (mutually adjusted model) | 0.98 (0.91–1.05) | 0.521 |
| Higher IC + Lower FI | Reference | — |
| Higher IC + Higher FI | 1.32 (1.13–1.54) | <0.001 |
| Lower IC + Lower FI | 2.03 (1.74–2.38) | <0.001 |
| Lower IC + Higher FI | 2.70 (2.35–3.09) | <0.001 |

## Supplementary Table 11. Sensitivity analysis excluding deaths occurring within the first year of follow-up (Model 3)

| **Variables** | **Effect estimate (95% CI)** | **P value** |
| --- | --- | --- |
| **All-cause mortality** |  |  |
| IC per 1-point increase (IC-only model) | 0.84 (0.82–0.86) | <0.001 |
| FI per 0.1 increase (FI-only model) | 1.19 (1.12–1.26) | <0.001 |
| IC per 1-point increase (mutually adjusted model) | 0.83 (0.81–0.85) | <0.001 |
| FI per 0.1 increase (mutually adjusted model) | 0.98 (0.91–1.05) | 0.508 |
| Higher IC + Lower FI | Reference | — |
| Higher IC + Higher FI | 1.24 (1.05–1.46) | 0.010 |
| Lower IC + Lower FI | 2.13 (1.79–2.54) | <0.001 |
| Lower IC + Higher FI | 2.50 (2.14–2.91) | <0.001 |

**Supplementary Table 12. Multinomial logistic regression accounting for death before the 2018 follow-up (Model 3)**

| **Variables** | **Incident disability vs alive without disability OR (95% CI)** | **P value** | **Death vs alive without disability OR (95% CI)** | **P value** |
| --- | --- | --- | --- | --- |
| **IC-only model** | | | | |
| IC, per 1-point increase | 0.79 (0.75-0.84) | <0.001 | 0.77 (0.73-0.81) | <0.001 |
| **FI-only model** | | | | |
| FI, per 0.1 increase | 1.84 (1.61-2.10) | <0.001 | 1.15 (1.02-1.28) | 0.019 |
| **Mutually adjusted IC-FI model** | | | | |
| IC, per 1-point increase | 0.84 (0.79-0.90) | <0.001 | 0.75 (0.71-0.78) | <0.001 |
| FI, per 0.1 increase | 1.60 (1.37-1.87) | <0.001 | 1.03 (0.97-1.10) | 0.627 |
| **Joint IC-FI categories** | | | | |
| Higher IC + Lower FI | Reference | - | Reference | - |
| Higher IC + Higher FI | 1.97 (1.48-2.63) | <0.001 | 1.28 (1.00-1.64) | 0.048 |
| Lower IC + Lower FI | 1.81 (1.03-3.20) | 0.041 | 3.02 (2.02-4.51) | <0.001 |
| Lower IC + Higher FI | 4.04 (2.54-6.43) | <0.001 | 3.33 (2.17-5.11) | <0.001 |

**Supplementary Table 13. Sensitivity analysis excluding self-rated health and self-rated quality of life from adjustment models (Model 3)**

| **Variables** | **Effect estimate (95% CI)** | **P value** |
| --- | --- | --- |
| **Incident disability** |  |  |
| IC per 1-point increase (IC-only model) | 0.81 (0.77–0.86) | <0.001 |
| FI per 0.1 increase (FI-only model) | 1.85 (1.61–2.13) | <0.001 |
| IC per 1-point increase (mutually adjusted model) | 0.88 (0.83–0.94) | <0.001 |
| FI per 0.1 increase (mutually adjusted model) | 1.66 (1.41–1.96) | <0.001 |
| Higher IC + Lower FI | Reference | — |
| Higher IC + Higher FI | 1.84 (1.38–2.46) | <0.001 |
| Lower IC + Lower FI | 1.44 (0.82–2.53) | 0.209 |
| Lower IC + Higher FI | 3.35 (2.11–5.31) | <0.001 |
| **All-cause mortality** |  |  |
| IC per 1-point increase (IC-only model) | 0.82 (0.81–0.84) | <0.001 |
| FI per 0.1 increase (FI-only model) | 1.35 (1.29–1.41) | <0.001 |
| IC per 1-point increase (mutually adjusted model) | 0.82 (0.80–0.84) | <0.001 |
| FI per 0.1 increase (mutually adjusted model) | 1.01 (0.95–1.07) | 0.815 |
| Higher IC + Lower FI | Reference | — |
| Higher IC + Higher FI | 1.27 (1.09–1.49) | 0.002 |
| Lower IC + Lower FI | 2.20 (1.88–2.58) | <0.001 |
| Lower IC + Higher FI | 3.01 (2.66–3.41) | <0.001 |

Note: All analyses used fully adjusted Model 3. For IC-only models, self-rated health and self-rated quality of life were removed from the original adjustment set; for FI-only, mutually adjusted IC-FI, and joint IC-FI models, self-rated quality of life was removed.

**Supplementary Table 14. Sensitivity analysis using multiple imputation by chained equations (Model 3)**

| **Variables** | **Effect estimate (95% CI)** | **P value** |
| --- | --- | --- |
| **Incident disability** |  |  |
| IC per 1-point increase (IC-only model) | 0.82 (0.77–0.87) | <0.001 |
| FI per 0.1 increase (FI-only model) | 1.88 (1.64–2.16) | <0.001 |
| IC per 1-point increase (mutually adjusted model) | 0.87 (0.81–0.92) | <0.001 |
| FI per 0.1 increase (mutually adjusted model) | 1.67 (1.42–1.96) | <0.001 |
| Higher IC + Lower FI | Reference | — |
| Higher IC + Higher FI | 1.89 (1.42–2.52) | <0.001 |
| Lower IC + Lower FI | 1.54 (0.90–2.64) | 0.114 |
| Lower IC + Higher FI | 3.40 (2.15–5.37) | <0.001 |
| **All-cause mortality** |  |  |
| IC per 1-point increase (IC-only model) | 0.83 (0.81–0.84) | <0.001 |
| FI per 0.1 increase (FI-only model) | 1.34 (1.29–1.40) | <0.001 |
| IC per 1-point increase (mutually adjusted model) | 0.82 (0.80–0.84) | <0.001 |
| FI per 0.1 increase (mutually adjusted model) | 1.01 (0.96–1.07) | 0.681 |
| Higher IC + Lower FI | Reference | — |
| Higher IC + Higher FI | 1.26 (1.09–1.47) | 0.002 |
| Lower IC + Lower FI | 2.21 (1.89–2.57) | <0.001 |
| Lower IC + Higher FI | 2.99 (2.64–3.38) | <0.001 |

Note: Multiple imputation by chained equations (MICE) was performed using 20 imputed datasets. Imputation was restricted to missing covariates; intrinsic capacity (IC), frailty index (FI), outcome variables, and survival time were not imputed. All analyses used fully adjusted Model 3, and estimates were pooled according to Rubin’s rules. Continuous IC was analyzed per 1-point increase and FI per 0.1 increase. Higher IC + Lower FI was the reference group for joint analyses. Incident disability was analyzed using logistic regression and all-cause mortality using Cox proportional hazards regression.

**Supplementary Table 15. Sensitivity analysis using modified Poisson regression for incident disability (Model 3)**

| **Variables** | **RR (95% CI)** | **P value** |
| --- | --- | --- |
| **IC and FI continuous analyses** |  |  |
| IC per 1-point increase (IC-only model) | 0.86 (0.83–0.90) | <0.001 |
| FI per 0.1 increase (FI-only model) | 1.52 (1.38–1.66) | <0.001 |
| IC per 1-point increase (mutually adjusted model) | 0.90 (0.87–0.94) | <0.001 |
| FI per 0.1 increase (mutually adjusted model) | 1.39 (1.25–1.55) | <0.001 |
| **Joint IC-FI categories** |  |  |
| Higher IC + Lower FI | Reference | — |
| Higher IC + Higher FI | 1.62 (1.30–2.01) | <0.001 |
| Lower IC + Lower FI | 1.48 (1.06–2.06) | 0.021 |
| Lower IC + Higher FI | 2.28 (1.76–2.95) | <0.001 |

Note: Modified Poisson regression with robust variance estimation was used to estimate risk ratios (RRs) and 95% confidence intervals (CIs) for incident disability. All analyses used fully adjusted Model 3. Continuous IC was analyzed per 1-point increase and FI per 0.1 increase. Higher IC + Lower FI was the reference group for joint analyses.

Figure S1 Flowchart of participant selection for the analyses of incident disability and all-cause mortality

**Figure S2. Restricted cubic spline curves for the associations of intrinsic capacity and frailty index with incident disability**

Note: Restricted cubic spline models were fitted using five knots at the 5th, 27.5th, 50th, 72.5th, and 95th percentiles of the exposure distributions. The median values of intrinsic capacity (IC; 7 points) and frailty index (FI; 0.17) were used as reference values (OR = 1.00). Models were adjusted using the covariates included in the corresponding fully adjusted Model 3. Solid lines indicate adjusted odds ratios (ORs), and shaded areas indicate 95% confidence intervals (CIs). For IC, P for overall <0.001 and P for nonlinearity = 0.267; for FI, P for overall <0.001 and P for nonlinearity = 0.153.

**Figure S3. Restricted cubic spline curves for the associations of intrinsic capacity and frailty index with all-cause mortality**

Note: Restricted cubic spline models were fitted using five knots at the 5th, 27.5th, 50th, 72.5th, and 95th percentiles of the exposure distributions. The median values of intrinsic capacity (IC; 7 points) and frailty index (FI; 0.17) were used as reference values (HR = 1.00). Models were adjusted using the covariates included in the corresponding fully adjusted Model 3. Solid lines indicate adjusted hazard ratios (HRs), and shaded areas indicate 95% confidence intervals (CIs). For IC, P for overall <0.001 and P for nonlinearity = 0.518; for FI, P for overall <0.001 and P for nonlinearity =0.112.
